# Knowledge of Fertility and Perspectives About Family Planning Among Female Physicians

**DOI:** 10.1001/jamanetworkopen.2022.13337

**Published:** 2022-05-18

**Authors:** Kathryn S. Smith, Jennifer B. Bakkensen, Anne P. Hutchinson, Elaine O. Cheung, Jessica Thomas, Veronika Grote, Patricia I. Moreno, Kara N. Goldman, Neil Jordan, Eve C. Feinberg

**Affiliations:** 1 Northwestern University Feinberg School of Medicine, Chicago, Illinois; 2 Division of Reproductive Endocrinology and Infertility, Department of Obstetrics and Gynecology at Northwestern University Feinberg School of Medicine, Chicago, Illinois; 3 Shady Grove Fertility in Philadelphia, Philadelphia, Pennsylvania; 4 Hinge, New York City, New York; 5 Preventive Medicine at Northwestern University, Feinberg School of Medicine, Chicago, Illinois; 6 Osher Center for Integrative Medicine at Northwestern University Feinberg School of Medicine, Chicago, Illinois; 7 Department of Public Health Sciences at the University of Miami Miller School of Medicine, Miami, Florida; 8 Institute for Public Health and Medicine-Center for Education in Health Sciences, Northwestern University Feinberg School of Medicine, Chicago, Illinois; 9 Center of Innovation for Complex Chronic Healthcare at the Edward J. Hines, Jr. VA Hospital, Hines, Illinois

## Abstract

**Importance:**

Pervasive gender disparities exist in medicine regarding promotion, achievement of academic rank, and appointment to leadership positions. Fertility and childbearing concerns may contribute to these disparities.

**Objective:**

To assess fertility knowledge and concerns and evaluate barriers to family building and impact on academic attrition reported by female physicians.

**Design, Setting, and Participants:**

This qualitative study used mixed methods; first, structured 1:1 interviews exploring fertility knowledge and family-building concerns were conducted among 16 female physicians between November 2019 and May 2020. Transcripts were coded in Dedoose and used to develop a survey instrument with subsequent pilot testing conducted among 24 female physicians between April 2020 and September 2020. Data analysis was performed from January 2021 to March 2021.

**Main Outcomes and Measures:**

Fertility knowledge, perceptions of peer and institutional support surrounding childbearing, factors contributing to delayed childbearing, and impact of family planning on career decisions.

**Results:**

Among 16 women who completed qualitative interviews, 4 (25%) were Asian, 1 (6%) was Black, 1 (6%) was multiracial, and 10 (63%) were White; mean (SD) age was 34.9 (4.0) years. Evaluation of fertility knowledge revealed 3 notable themes: (1) inadequate formal fertility education, (2) informal learning through infertility experiences of patients, peers, or personal struggles, and (3) desire to improve medical education through early introduction and transparent discussions about infertility. Exploration of childbearing concerns similarly revealed several salient themes: (1) high incidence of delayed childbearing, (2) perceived lack of peer and administrative support, and (3) impact of family building on career trajectory. These themes were borne out in pilot testing of the survey instrument: of 24 female physicians (7 Asian women [27%], 1 Black woman [4%], 1 Hispanic or Latinx woman [4%], 1 multiracial woman [4%], 15 White women [58%]; mean [SD] age, 36.1 [6.7] years), 17 (71%) had delayed childbearing and 16 (67%) had altered their career for family-building reasons.

**Conclusions and Relevance:**

Qualitative interviews identified fertility and family building concerns among female physicians and were used to develop a tailored survey for women in medicine. These findings suggest that female physicians may delay childbearing and make substantial accommodations in their careers to support family building. A large-scale national survey is needed to better characterize the unique fertility, childbearing, and parenting needs of women in academic medicine to better understand how these concerns may contribute to academic attrition.

## Key Points

**Question:**

What are the fertility and family planning concerns of female physicians, and how do they affect women’s career trajectory in medicine?

**Findings:**

This qualitative study with structured 1:1 qualitative interviews with 16 female physicians identified fertility and family-building concerns that were used to develop and pilot test a survey for women in medicine. Results from the pilot survey of 24 female physicians found that 71% had delayed childbearing and 67% had altered their career for family-building reasons.

**Meaning:**

These findings suggest that female physicians may delay childbearing and make substantial accommodations in their careers to support family building.

## Teaser

This qualitative study assesses fertility knowledge and concerns and evaluates barriers to family building and impact on academic attrition reported by female physicians.

## Introduction

Despite closing the gender gap in matriculation into medical school and representation among academic medicine faculty, women remain less likely to hold academic leadership positions and more likely to leave academic medicine compared with men.^1,2,3^ According to 2020 data from the Association of American Medical Colleges (AAMC), women account for 43% of medical school faculty but only 21% of department chairs and 19% of medical school deans.^4^ These disparities do not appear to be improving over time despite heightened awareness of the gender gap, as evidenced by a 2020 cohort study of physicians during a 35-year period, which found that women were less likely than men to be promoted to the title of associate professor, professor, or department chair, with no narrowing of the gap over time.^5^

Although the reasons underlying these disparities are complex, fertility and family-building concerns among women in medicine may be contributing to academic attrition. Female physicians are more likely to delay childbearing compared with nonphysicians,^6,7,8^ which may contribute to the high rates of infertility observed among female physicians.^9,10,11,12,13^ Furthermore, the decision to delay childbearing is commonly underinformed. Studies have reported low fertility knowledge among US women and medical trainees.^14,15^ Despite this knowledge gap, little research has been done on the role of medical education in providing trainees with information about age-related fertility decline and fertility preservation.

In addition to delaying childbearing, female physicians may face inadequate support when ready to grow their family.^16,17,18,19,20,21,22^ This perceived lack of support may cause women to alter their careers to accommodate childbearing. In a survey of 600 female physicians, more than 70% reported that having children had influenced their career.^9^ These findings highlight a need to better understand how experiences of family building among women in medicine may influence career trajectory, and to explore the extent to which family-building concerns contribute to the persistent gender disparities within academic medicine.

The objectives of this study were to (1) qualitatively assess fertility knowledge and family-building concerns among female physicians, and (2) develop a survey instrument to characterize the unique needs of women in medicine surrounding fertility and family planning, and how these needs may impact career trajectory.

## Methods

### Sample Recruitment

This qualitative study was approved by the Northwestern University institutional review board and all participants provided written informed consent prior to participation. The study followed the Consolidated Criteria for Reporting Qualitative Research (COREQ) reporting guideline.^23^

Participants were recruited in-person at a conference for women in medicine at Northwestern University Feinberg School of Medicine in October 2019 and through social media via Facebook’s Physician Moms Group between October 2019 and May 2020 using convenience sampling. All women completed a screening questionnaire with information including age, self-reported ethnicity, and sexual orientation in order to obtain basic demographical data. Female physicians who graduated from medical school between 1999 and 2019 having a current or former position in academic medicine were eligible to participate in interviews. Female physicians who were eligible to participate in the interviews were invited to participate via email by E.C. In the email invitation, participants were informed about the aim of the research and the researchers’ interest in the topic. Pilot survey recruitment included women who completed the screening questionnaire mentioned previously but did not participate in qualitative interviews. Pilot survey participation was limited to women with a current or former position in academic medicine. Female physicians who were eligible to participate in the pilot survey were invited to participate via email by E.C. and were explained the aims of the research.

### Interview Format

Interviews were conducted in-person in interview rooms at Northwestern University or remotely via video conference from November 2019 through May 2020. Interviews were conducted using a semistructured interview guide and lasted approximately 1 hour (eAppendix 1 in the Supplement). Each participant was interviewed only once, and each participant was assigned a record number to maintain deidentified status. The interviews were conducted by a female, PhD-level psychology researcher (E.C.) who was employed as a research assistant professor at Northwestern University with prior qualitative research training. For each interview, one additional female researcher was present and took notes during the interviews (K.S., J.T., V.G.). K.S. was a medical student at Northwestern University, J.T. was a masters-level, licensed clinical social worker who was employed as a research project coordinator at Northwestern University, and V.G. was a bachelors-level research project coordinator at Northwestern University. No relationship existed between the researchers and any participant in advance. There were no nonparticipants present for the interviews. Open-ended questions assessed participants’ knowledge of age-related fertility decline, sources of fertility knowledge, and recommendations for improving medical education in fertility and family planning. Participants were asked about their intentions for parenthood, desired family size, considerations regarding the timing of fertility/reproduction and childbearing, mechanisms of support within the workplace, policies related to family building, and support outside the workplace (eg, from partner). Finally, interview questions inquired about knowledge of the effects of age on fertility and assisted reproductive technologies (ART), influence of fertility concerns on career decisions, and awareness and attitudes surrounding ART. Interviews were conducted until data saturation was reached. Transcripts were not returned to interviewees for comment or correction.

### Qualitative Analysis

All sessions were audio recorded, transcribed, and deidentified for analysis using Dedoose. The study team used a constant comparative approach to analyze the data from the interview transcripts.^24,25^ To develop the codebook, members of the study team each independently read 1 interview to identify common themes that appeared within the text and develop an initial set of codes. Based on team review and interpretation, the codebook was iteratively revised and refined. The study team continued to code several transcripts, until the team reached a consensus on a set of working codes. Once the coding was finalized, the study team coded all transcripts, including recoding the initial interviews used to create the codebook. Each transcript was coded independently by 2 team members. Eight study team members in total coded the transcripts. Discrepancies were resolved through discussion until consensus was reached by the study team, and necessary adjustments were made to the final coded version of each transcript. The coding included 5 major codes: (1) medical education/awareness of the role of age in fertility, (2) financial cost and insurance coverage, (3) workplace support, (4) support outside of work, and (5) influence of fertility and family planning on career path (eAppendix 2 in the Supplement). Sorting for data reduction and thematic structuring of the themes and subthemes were performed through virtual sessions between the study team. Data analysis was organized by codes with frequency of responses used to generate themes and subthemes as agreed upon by the research team through consensus. Based on group review and interpretation, analysis of the interviews led to a coding tree with 2 themes and 6 subthemes derived from the data (Table 1). Data analysis was performed from January 2021 to March 2021.

**Table 1.** Themes and Subthemes Regarding Fertility Knowledge Among Women in Medicine That Arose From Qualitative Interviews With 16 Physicians

Theme	Subthemes	Exemplary quotations^a^
Fertility knowledge	Inadequate formal education	“The majority of what I learned about from a fertility standpoint was basic sort of how the reproductive system works. ... I do think that aging and fertility is something that we were told about, but infertility in women under 35 is something we didn’t hear about.” (1010)
		“I think it [age and infertility] was briefly touched upon in medical school during my OB/GYN rotation, but not much more than at age 35 your risk for Down syndrome goes up markedly. … I wouldn’t say that it was really emphasized at all. I don’t think that I had any sort of opportunity to go to an REI [reproductive endocrinology] clinic or had any exposure to that in medical school.” (1001)
		“Learning in medical school is what I would call outdated, maybe, statistics on fertility, drop off for fertility … based pretty much on some older data that was always associated with fear and panic and you’re never going to be able to get pregnant. … We certainly had no instruction whatsoever on family planning.” (1015)
	Reliance on informal sources of education	“There were a couple of times in my medical school career as I was thinking about different career paths where I talked to mentors that I had known for a long time and that I trusted. I asked them for that kind of feedback about how that particular specialty or particular place, how that would impact my family life and my desired lifestyle.” (1013)
		“I learned through talking to my coworkers. … I went through a miscarriage and now infertility and I think that I was able to feel very supported in it because a lot of my colleagues are also very open about what they’ve been through with miscarriage, infertility, and pregnancy.” (1004)
	Improving medical education for medical trainees	“I would add it to your residency orientation, because I think you're capturing people in their 20s for the most part. I think that’s an ideal age, and I think that if people have it in the back of their mind, they are going to be more cognizant …” (1006)
		“Medical school is when people are still considering different fields and telling us different fields may affect their fertility choices and options … so, before you’re in the time where you’re really thinking about starting a family, to have the information ahead of time would be good.” (1008)
Barriers to family building and impact on career trajectory	Delayed childbearing	“Ideally, I always wanted to have a family, but I was divorced at 35 and I had more than the average amount of projects and jobs, postdoc residency and fellowship, all these things at once, trying to get grants, was too much.” (1015)
		“I still feel like there’s still a lot of pressure to wait and this is your time for training, you could do that stuff later. I see it would culturally be very difficult to get people onboard with why a student or why a trainee might not want to wait.” (1007)
	Insufficient resources and support	“The culture of constant self-sacrifice … like coming back to work early, and not taking the full maternity leave.” (1002)
		“… [I]t’s like are we going to be punished if you take those full 12 weeks of maternity leave? Is your salary going to go down? Are you taking a pay cut? I don’t think it’s been explained well.” (1001)
		“I think that it would also be helpful for medical students and trainees to know what their options are, what insurance covers. ... It wasn’t even touched at my orientation here as an attending.” (1001)
		“I have seen a lot of my friends try really hard and almost kill themselves with excessive amount of service time just so that they don’t have to unload any of their workload on to somebody else unnecessarily.” (1001)
	Altering career path to accommodate childbearing	“It’s something I really love and enjoy doing but it’s not the only thing and having kids is something very important to me … if I felt like my current position was taking away from that I’d be happy to make accommodations or even leave medicine.” (1001)
		“When it comes to maternity leave or what we want to change, if I want to change my schedule, even just to go to a school program or something, I get to make those decisions. Whereas in academic medicine, it was a few more hoops to jump through to get hours off.” (1016)
		“I think there’s actually people who choose not to go into medicine for this reason. If we’re not addressing it, we are missing out on women who might want to be doctors but are right about, that they can’t have a family, or that they don’t want to wait until they’re out of training.” (1007)

^a^ Numbers in parentheses indicate the record number assigned to each participant of the qualitative interviews.

### Survey Development

Data from qualitative interviews were used to develop a survey (eAppendix 3 in the Supplement) assessing demographics, partner status, career path, family planning, childbearing, and infertility. The survey consisted of multiple-choice questions and Likert-type scales. A panel of psychologists and reproductive endocrinologists assessed the survey for content validity. The survey was pilot tested among female physicians. The pilot survey included open-ended questions probing participants on the clarity, appropriateness, and comprehensiveness of the survey items. The survey items were iteratively revised based on participants’ feedback.

## Results

Among the 43 female physicians who completed the screening questionnaire, 4 (9.3%) were recruited from the women in medicine conference, 13 (30.2%) were recruited from the Facebook Physician Moms Group, 16 (37.2%) were referred to participate in the study from a colleague/friend who heard about the study, and 10 (23.2%) saw the study shared on other social media platforms.

### Qualitative Interviews

Among 16 women who participated in qualitative interviews, 4 (25%) were Asian, 1 (6%) was Black, 1 (6%) was multiracial, and 10 (63%) were White; there were 13 attending physicians, 1 resident physician, 1 clinical fellow, and 1 pharmaceutical medical director; mean (SD) age was 34.9 (4.0) years. Most participants were heterosexual (n = 15; 93.8%), married/partnered (n = 15; 93.8%), and had children (n = 9; 56.3%). No participants refused to participate or dropped out. Respondent demographics and characteristics are outlined in Table 2.

**Table 2.** Demographic Characteristics From Qualitative Interviews and Pilot Survey Assessing Fertility Knowledge and Family Building Concerns Among Women in Medicine

**Characteristic**	**Participants, No. (%)**	
	**Qualitative interviews (N = 16) **	**Pilot survey (N = 24)**
Age, mean (SD)	34.9 (4.0)	36.1 (6.7)
Ethnicity		
Asian	4 (25)	7 (27)
Black	1 (6)	1 (4)
Hispanic or Latinx	0	1 (4)
Multiracial	1 (6)	1 (4)
White	10 (63)	15 (58)
Not listed	0	1 (4)
Sexual orientation		
Heterosexual	15 (94)	21 (88)
Gay or lesbian	1 (6)	1 (4)
Bisexual	0	1 (4)
Not listed	0	1 (4)
Relationship status		
Married/partnered	15 (94)	22 (92)
Single	1 (6)	2 (8)
Current position/occupation		
Resident	1 (6)	3 (13)
Fellow	1 (6)	3 (13)
Attending	13 (81)	18 (75)
Other	1 (6)	0
Current practice setting^a^		
Private	3 (19)	4 (21)
Academic	9 (56)	12 (63)
Community	0	3 (16)
Other	1 (6)	2 (7)
Planned practice setting^b^		
Private	1 (6)	1 (13)
Academic	1 (6)	5 (63)
Community	0	2 (25)
Income, $		
<99 000	1 (6)	1 (4)
100 000-250 000	10 (63)	11 (46)
250 000-500 000	3 (19)	6 (25)
>500 000	2 (13)	5 (21)
Do you have children?		
Yes	8 (50)	16 (67)
No	8 (50)	8 (33)
Do you plan to have additional children?^c^		
Yes	7 (88)	7 (44)
No	1 (13)	5 (31)
Undecided	0	4 (25)

^a^ Among attendings (qualitative interviews: n = 13; pilot survey: n = 18).

^b^ Among residents and fellows (qualitative interviews: n = 2; pilot survey: n = 6).

^c^ Among respondents with children (qualitative interviews: n = 8; pilot survey: n = 16).

#### Theme 1: Fertility Knowledge

Interviews revealed 3 major subthemes regarding fertility knowledge: (1) inadequate formal education, (2) reliance on informal education, and (3) a desire for improved formal medical education (eAppendix 4 in the Supplement). Exemplary quotations from each subtheme are outlined in Table 1.

##### Subtheme 1: Inadequate Formal Training

All participants cited inadequate medical school education surrounding fertility. Fifteen participants recalled learning about age-related fertility decline but remembered little instruction about family planning or assisted reproductive technologies (ART). Participants also expressed that information they received in medical school was outdated or misleading. For example, one woman recalled being taught that fertility decline begins at age 35 and felt this led to a false sense of security among women in their early thirties. Others recalled that fertility was presented in a way that fostered fear, without education about fertility preservation.

“Learning in medical school is what I would call outdated ... based pretty much on some older data and was always associated with fear and panic and you’re never going to be able to get pregnant …We certainly had no instruction whatsoever on family planning” (participant 1015).

##### Subtheme 2: Reliance on Informal Education 

Citing inadequate formal education, participants reported learning about infertility informally through the experiences of patients, peers, or personal experiences. Notably, one participant described how she realized the high prevalence of infertility among female physicians through speaking with peers and cited these interactions as critical support as she faced her own infertility diagnosis.

“I learned through talking to my coworkers … I went through a miscarriage and now infertility and I think that I was able to feel very supported in it because a lot of my colleagues are also very open about what they’ve been through with miscarriage, infertility, and pregnancy” (participant 1004).

##### Subtheme 3: Desire for Improved Formal Medical Education 

Overall, participants endorsed a need for improved formal fertility education. Many believed that information about age-related fertility decline and fertility preservation options should be presented early in medical training, with some specifying that this information be conveyed before medical students have selected their intended specialty to allow fertility considerations to play a role in specialty choice.

“Medical school is when people are still considering different fields and telling us different fields may affect their fertility choices and options … so, before you’re in the time where you’re really thinking about starting a family, to have the information ahead of time would be good” (participant 1008).

Participants supported facilitating open conversations among physicians about work-life balance, family building, and infertility, as well as incorporating dialogue with trusted colleagues and mentors, especially among those with similar career paths and goals.

#### Theme 2: Barriers to Family Building and Impact on Career Trajectory

Interviews revealed 3 major subthemes surrounding barriers to family building, including (1) delayed childbearing, (2) insufficient resources and support, and (3) impact of childbearing on career trajectory. Exemplary quotes for each theme and subtheme are outlined in Table 1.

##### Subtheme 1: Delayed Childbearing 

Although participants were aware of age-related fertility decline, 14 of 16 shared that they had delayed or were currently delaying childbearing. Ten cited barriers in training and/or work environment as the primary cause of this delay, whereas 4 cited a lack of a partner as the primary reason.

##### Subtheme 2: Insufficient Resources and Support 

Participants discussed that insufficient mechanisms of support, including workplace culture and policies, played an important role in their decision to delay childbearing. Without knowledge of certain benefits, or belief that these benefits were insufficient, women felt ill-equipped to start a family. They noted a lack of formal introduction to benefits, such as parental leave and childcare, and little guidance in navigating fertility preservation options. Consequently, many women felt their decisions surrounding childbearing were underinformed. To prevent these feelings of uncertainty, interviewees recommended having transparent discussions with trainees on fertility, family planning considerations, and institution-specific benefits (eg, parental leave, fertility-preservation benefits, and infertility coverage).

“I think that it would also be helpful for medical students and trainees to know what their options are, what insurance covers ... It wasn’t even touched at my orientation here as an attending” (participant 1001).

Participants also emphasized the importance of changing the broader culture within medicine, citing a lack of support from physician peers and leadership. Two women perceived a gender difference in leadership support and stressed the importance of having women in leadership positions, especially when creating policies pertaining to pregnancy, parental leave, and parenthood.

##### Subtheme 3: Impact of Childbearing on Career Trajectory

 Interviews demonstrated that family planning affected career trajectory. Some women had specifically chosen their specialty based on flexibility for family planning. One woman had left academic medicine for private practice to increase autonomy over her schedule. Women who experienced stressors such as lack of schedule flexibility or social or institutional support were more likely to report symptoms of burnout and, in some cases, considered leaving medicine entirely. Thirteen women stated they would reduce their clinical time for additional flexibility for childbearing, and 2 women stated they would consider changing specialties or leaving medicine altogether to accommodate pregnancy and parenthood. One woman had left clinical medicine owing to a combination of these stressors.

“It’s something I really love and enjoy doing but it’s not the only thing and having kids is something very important to me … if I felt like my current position was taking away from that I’d be happy to make accommodations or even leave medicine” (participant 1001).

### Pilot Survey

Among the 24 women who completed the pilot survey between January 2020 and February 2020 (eAppendix 3 in the Supplement), 7 (27%) were Asian, 1 (4%) was Black, 1 (4%) was Hispanic or Latinx, 1 (4%) was multiracial, and 15 (58%) were White; mean (SD) age was 36.1 (6.7) years. Respondent demographics and characteristics are outlined in Table 2.

Most respondents (n = 14, 58%) correctly identified that fertility declines most precipitously after the age of 35 years. Although 21 respondents (88%) correctly identified that fecundability among women aged 43 to 45 years is less than 5%, 15 (63%) overestimated the ability of ART to overcome age-related fertility decline within this age group. Using a 5-point Likert-type scale (1 = not at all to 5 = extremely), participants rated the information sources they relied on to learn about the role of age and fertility. As seen in Table 3, respondents reported moderate reliance on the internet and personal experiences to learn about the role of age and fertility, with 21 (88%) of women reporting at least moderate reliance on the internet (≥3 out of 5) and 12 (50%) reporting at least moderate reliance on personal experiences. As seen in Table 3, respondents reported relatively low reliance across all information sources for learning about egg freezing, with 14 (58%) reporting at least moderate reliance on the internet for information on egg freezing.

**Table 3.** Female Physicians’ (N = 24) Self-reported Reliance on Information Sources to Learn About the Role of Age in Fertility and Egg Freezing

Information source	Self-reported reliance on information sources, mean (SD)^a^	
	Role of age in fertility	Egg freezing
Internet	3.08 (1.25)	2.21 (1.25)
Your own personal experiences	3.00 (1.32)	2.08 (1.56)
Experiences of family and friends	2.58 (1.25)	1.92 (1.21)
Formal education in medical school	2.50 (1.29)	1.58 (1.06)
Your own health care provider	2.50 (1.29)	1.79 (1.29)
Experiences of physician colleagues	2.29 (1.08)	1.96 (1.16)
Experiences of patients	2.17 (1.34)	1.71 (1.08)
Educational content from social media	1.79 (1.06)	1.71 (1.04)
News outlets	1.79 (1.06)	1.54 (0.93)
Individual posts from social media	1.75 (1.08)	1.54 (0.93)
Television/film	1.58 (0.97)	1.50 (0.89)

^a^ Female physicians’ self-reported reliance on information sources to learn about the role of age in fertility and egg freezing (1 = not at all to 5 = extremely).

Seventeen respondents (71%) reported delaying childbearing due to medical training or choice of specialty. Again, using the same 5-point Likert-type scale, participants rated how influential a variety of factors were in influencing their decision to delay childbearing. As seen in Table 4, respondents rated that their decision to delay childbearing was moderately impacted by factors including lack of flexibility in work schedule, stress, and lack of time, with most women citing lack of flexibility in work schedule (67%; n = 16), stress (67%; n = 16) and lack of time 63% (n = 15) as having at least moderate influence in their decision to delay childbearing. Most respondents (71%; n = 17) were at least moderately concerned about how the length of medical training would impact family building (≥3 out of 5; mean [SD], 3.08 [0.97]). Furthermore, 67% (n = 16) had altered their career in some way to accommodate childbearing or parenthood: 29% (n = 7) did not take opportunities for career advancement, 21% (n = 5) had chosen a different specialty, and 17% (n = 4) had changed their work setting (academic vs private practice).

**Table 4.** Female Physicians’ (N = 24) Ratings of the Extent to Which Certain Factors Influenced Their Decisions About the Timing of Childbearing

**Factors**	**Influence on childbearing decision, mean (SD)** ^a^
Lack of flexibility in schedule	3.29 (1.23)
Stress	3.29 (1.20)
Lack of time	3.25 (1.15)
Financial strain	2.79 (1.10)
Concern about burdening colleagues	2.54 (1.10)
Reputational concerns/stigma	2.52 (1.20)
Lack of social support nearby	2.35 (1.03)
Not ready for children	2.29 (1.23)
Lack of support from colleagues	2.26 (1.18)
Lack of romantic partner	2.25 (1.70)
Lack of support from leadership	2.04 (1.33)

^a^ Female physicians’ ratings of the extent to which certain factors influenced their decisions about the timing of childbearing (1 = not at all to 5 = extremely).

There were 11 respondents (46%) who had personally experienced infertility, of which 7 (64%) had used IVF for conception. There were 10 respondents (42%) who had considered fertility preservation, of which 8 (80%) had previously sought consultation, including 5 respondents who had frozen eggs and 2 respondents who were in the process of freezing (or planning to freeze) their eggs. As seen in Table 5, respondents reported that age, relationship status, financial cost, and insurance coverage had moderately high influence on their decision of whether to pursue egg/embryo freezing; 10 respondents (42%) reported that they would pursue egg freezing if it were a covered benefit, but only 6 (25%) reported knowing that egg freezing was, in fact, covered by health insurance, whereas 13 (54%) did not know the status of their coverage.

**Table 5.** Female Physicians’ (N = 10) Ratings of the Extent to Which Certain Factors Influenced Their Decision Whether to Freeze Their Eggs/Embryos

Factors	Influence on decision to freeze eggs/embryos, mean (SD)^a^
Age	3.89 (1.05)
Relationship status	3.75 (1.39)
Financial cost	3.56 (1.01)
Insurance coverage	3.00 (1.63)
Burden	2.89 (1.36)
Effectiveness/likelihood of success	2.80 (1.40)
Time commitment	2.60 (1.51)
Potential risks associated with procedure	2.50 (1.35)

^a^ Among female physicians who had considered fertility preservation, respondents’ ratings of the extent to which certain factors influenced their decision whether to freeze their eggs/embryos (1 = not at all to 5 = extremely).

## Discussion

Contrary to previous studies that found low fertility knowledge among medical trainees,^14,15^ our results suggest that women in medicine are aware of the impact of age on fertility. Despite this knowledge, most women in our study had delayed childbearing and a large proportion had personally experienced infertility, consistent with previous studies suggesting high rates of infertility among female physicians.^9^ Furthermore, women tend to overestimate the ability of ART to overcome age-related fertility decline. These data highlight the need for improvement in education about the limitations of currently available technologies to treat infertility and, more specifically, to overcome ovarian aging. Participants felt that formal training about the impact of age on fertility and the high rates of infertility among female physicians should occur early in trainees’ education to allow for informed decision-making in choice of specialty and family-building plans.

In recent years, oocyte and embryo cryopreservation have emerged as a means of increasing reproductive options.^26,27^ In line with prior research,^28,29,30^ women in the present study demonstrated high interest in fertility preservation. Nevertheless, most women received information on fertility preservation from informal sources (eg, the internet). Options for fertility preservation should accompany any discussion of fertility in the formal medical school curriculum. Furthermore, most pilot survey respondents did not know if egg freezing was covered by their health insurance plan. Cost is a well-documented concern of women contemplating elective egg freezing.^31^ Therefore, residency and fellowship training programs should provide their trainees with information on institution-specific insurance coverage and estimated costs of fertility preservation.^32^

In addition to highlighting a need to improve fertility education, our results suggest that women in medicine make substantial adjustments to their career trajectory to accommodate family building. Strikingly, 29% of survey respondents declined opportunities for career advancement, 21% chose a different specialty, and 17% changed their work setting from academic to private practice to accommodate having children. These results are alarming, particularly in light of known gender disparities that exist within academic medicine in time-to-promotion, achievement of academic rank, and appointment to leadership positions. It is, therefore, imperative to understand and ultimately address the family-building concerns specific to women in academic medicine. In the present study, commonly cited concerns pertained to unsupportive workplace policies and culture. Women cited a lack of support from physician peers and leadership, particularly related to taking time off for pregnancy, maternity leave, infertility treatments, or parenting obligations.^33,34,35,36,37,38,39,40,41^ The marked prevalence of fertility and family-building concerns in the current study suggest that facilitating open communication and peer support between physicians may be an important step in this culture shift.

Considering the current focus within academic medicine on wellness and female physician retention, our study addresses critical issues impacting women in medicine. The pilot survey is an initial effort to characterize the unique fertility needs and family planning concerns of women in medicine. Future directions include a larger national survey which will allow us to better describe factors that were not fully evaluated in this study due to its small sample size. National data will allow us to stratify findings based on level of training, setting of practice, and specialty to determine if substantial variations in experience exist based on these factors.

### Strengths and Limitations

This study had some strengths. The use of semistructured qualitative interviews in this study provided a narrative account of women’s experience with fertility in academic medicine and allowed for in-depth exploration of a nuanced topic. Questions in the pilot survey were based on themes that arose from the qualitative interviews and, therefore, addressed topics most pertinent to female physicians. Evaluation of the survey instrument by a panel of psychologists and reproductive endocrinologists ensured content validity, and subsequent evaluation among female physicians ensured appropriate response distribution, clarity, sensitivity, and depth.

This study also had limitations. For example, it was limited by its potential for self-selection bias. Specifically, personal experiences with infertility or childbearing may have motivated participation in the qualitative interviews or survey. In addition, our study sample was primarily recruited from a women-in-medicine conference and social media via a Facebook Physician Moms group, which may not be a representative sample of all female physicians. Furthermore, our study sample primarily consisted of attending physicians, which precluded us from exploring differences based on career stage. Although conclusions from the pilot survey are limited by its small sample size, our preliminary data yielded striking results and has set the stage for distribution among a national sample to obtain more robust data about fertility and family-building concerns among female physicians.

## Conclusions

Persistent gender disparities exist in leadership and faculty rank in academic medicine despite equal matriculation into medical school. Results of this study suggest that women may alter their career trajectory to accommodate family building. A large-scale national survey is needed to better characterize the unique fertility, childbearing, and parenting needs among women in academic medicine to understand and, ultimately, address the gender gap.

## Notes

Supplement. **eAppendix 1.** Qualitative Interview Guide **eAppendix 2.** Qualitative Interview Codebook **eAppendix 3.** Pilot Survey **eAppendix 4.** Themes and Subthemes That Arose From Qualitative Interviews With 16 Women Physicians

## References

[1] Colleges AoAM. Table 8: U.S. Medical School Faculty by Sex and Race/Ethnicity, 2018. Accessed April 13, 2022. https://www.aamc.org/download/495038/data/18table8.pdf

[2] Thibault GE. Women in academic medicine. Acad Med. 2016;91(8):1045-1046. doi:10.1097/ACM.0000000000001273 27306968

[3] Carr PL, Raj A, Kaplan SE, Terrin N, Breeze JL, Freund KM. Gender differences in academic medicine: retention, rank, and leadership comparisons from the National Faculty Survey. Acad Med. 2018;93(11):1694-1699. doi:10.1097/ACM.0000000000002146 29384751 PMC6066448

[4] Association of American Medical Colleges. 2020 U.S. Medical school faculty. Accessed April 13, 2022. https://www.aamc.org/data-reports/faculty-institutions/interactive-data/2020-us-medical-school-faculty

[5] Richter KP, Clark L, Wick JA, . Women physicians and promotion in academic medicine. N Engl J Med. 2020;383(22):2148-2157. doi:10.1056/NEJMsa1916935 33252871

[6] Cusimano MC, Baxter NN, Sutradhar R, . Delay of pregnancy among physicians vs nonphysicians. JAMA Intern Med. 2021;181(7):905-912. doi:10.1001/jamainternmed.2021.1635 33938909 PMC8094034

[7] Rangel EL, Castillo-Angeles M, Easter SR, . Incidence of infertility and pregnancy complications in US female surgeons. JAMA Surg. 2021;156(10):905-915. doi:10.1001/jamasurg.2021.3301 34319353 PMC9382914

[8] Stack SW, Jagsi R, Biermann JS, . Childbearing decisions in residency: a multicenter survey of female residents. Acad Med. 2020;95(10):1550-1557. doi:10.1097/ACM.0000000000003549 32568852

[9] Stentz NC, Griffith KA, Perkins E, Jones RD, Jagsi R. Fertility and childbearing among American female physicians. J Womens Health (Larchmt). 2016;25(10):1059-1065. doi:10.1089/jwh.2015.5638 27347614

[10] Wesselink AK, Rothman KJ, Hatch EE, Mikkelsen EM, Sørensen HT, Wise LA. Age and fecundability in a North American preconception cohort study. Am J Obstet Gynecol. 2017;217(6):667.e1-667.e8. doi:10.1016/j.ajog.2017.09.002 28917614 PMC5712257

[11] Chandra A, Copen CE, Stephen EH. Infertility and impaired fecundity in the United States, 1982-2010: data from the National Survey of Family Growth. Natl Health Stat Report. 2013;(67):1-18, 1, 19. 24988820

[12] Hassold T, Hunt P. To err (meiotically) is human: the genesis of human aneuploidy. Nat Rev Genet. 2001;2(4):280-291. doi:10.1038/35066065 11283700

[13] Durfey SNM, White J, Adashi EY. Pregnancy and parenting in medical school: highlighting the need for data and support. Acad Med. 2021;96(9):1259-1262. doi:10.1097/ACM.0000000000003988 33570853

[14] Roberts LM, Kudesia R, Zhao H, Dolan S, Rose M. A cross-sectional survey of fertility knowledge in obstetrics and gynecology residents. Fertil Res Pract. 2020;6(1):22. doi:10.1186/s40738-020-00091-2 33292597 PMC7724860

[15] Yu L, Peterson B, Inhorn MC, Boehm JK, Patrizio P. Knowledge, attitudes, and intentions toward fertility awareness and oocyte cryopreservation among obstetrics and gynecology resident physicians. Hum Reprod. 2016;31(2):403-411. 26677956 10.1093/humrep/dev308

[16] Chesak SS, Yngve KC, Taylor JM, Voth ER, Bhagra A. Challenges and solutions for physician mothers: A critical review of the literature. Mayo Clin Proc. 2021;96(6):1578-1591. doi:10.1016/j.mayocp.2020.10.008 33840524

[17] Freeman G, Bharwani A, Brown A, Ruzycki SM. Challenges to navigating pregnancy and parenthood for physician parents: a framework analysis of qualitative data. J Gen Intern Med. 2021;36(12):3697-3703. doi:10.1007/s11606-021-06835-0 33959880 PMC8642566

[18] Treister-Goltzman Y, Peleg R. Female physicians and the work-family conflict. Isr Med Assoc J. 2016;18(5):261-266. 27430080

[19] La Torre G, Grima D, Romano F, Polimeni A. Perceived work ability and work-family conflict in healthcare workers: an observational study in a teaching hospital in Italy. J Occup Health. 2021;63(1):e12271. doi:10.1002/1348-9585.12271 34535041 PMC8448582

[20] Ruzycki SM, Freeman G, Bharwani A, Brown A. Association of physician characteristics with perceptions and experiences of gender equity in an academic internal medicine department. JAMA Netw Open. 2019;2(11):e1915165-e1915165. doi:10.1001/jamanetworkopen.2019.15165 31722028 PMC6902791

[21] Harris CE, Clark SD, Chesak SS, . GRIT: women in medicine leadership conference participants’ perceptions of gender discrimination, disparity, and mitigation. Mayo Clin Proc Innov Qual Outcomes. 2021;5(3):548-559. doi:10.1016/j.mayocpiqo.2021.02.007 34195547 PMC8240154

[22] Aghajanova L, Hoffman J, Mok-Lin E, Herndon CN. Obstetrics and gynecology residency and fertility needs. Reprod Sci. 2017;24(3):428-434. doi:10.1177/1933719116657193 27368879

[23] Tong A, Sainsbury P, Craig J. Consolidated criteria for reporting qualitative research (COREQ): a 32-item checklist for interviews and focus groups. Int J Qual Health Care. 2007;19(6):349-357. doi:10.1093/intqhc/mzm042 17872937

[24] Strauss A, Corbin JM. Basics of qualitative research: Grounded theory procedures and techniques. Sage Publications, Inc; 1990.

[25] Cohen D, Crabtree B. Qualitative Research Guidelines Project: guidelines for designing, analyzing and reporting qualitative research. July 2006. Accessed April 13, 2022. http://qualres.org/HomeGuid-3868.html

[26] Dondorp W, de Wert G, Pennings G, ; ESHRE Task Force on Ethics and Law. Oocyte cryopreservation for age-related fertility loss. Hum Reprod. 2012;27(5):1231-1237. doi:10.1093/humrep/des029 22357771

[27] Cobo A, Garcia-Velasco JA, Domingo J, Remohí J, Pellicer A. Is vitrification of oocytes useful for fertility preservation for age-related fertility decline and in cancer patients? Fertil Steril. 2013;99(6):1485-1495. doi:10.1016/j.fertnstert.2013.02.050 23541405

[28] Wang A, Herndon CN, Mok-Lin E, Aghajanova L. Infertility, fertility preservation, and access to care during training: a nationwide multispecialty survey of united state residents and fellows. Journal of Fertility Preservation. 2021;2. doi:10.32371/jfp/246110

[29] Ikhena-Abel DE, Confino R, Shah NJ, . Is employer coverage of elective egg freezing coercive?: a survey of medical students’ knowledge, intentions, and attitudes towards elective egg freezing and employer coverage. J Assist Reprod Genet. 2017;34(8):1035-1041. doi:10.1007/s10815-017-0956-9 28577184 PMC5533686

[30] Merchant SJ, Hameed SM, Melck AL. Pregnancy among residents enrolled in general surgery: a nationwide survey of attitudes and experiences. Am J Surg. 2013;206(4):605-610. doi:10.1016/j.amjsurg.2012.04.005 23200987

[31] Katz P, Showstack J, Smith JF, . Costs of infertility treatment: results from an 18-month prospective cohort study. Fertil Steril. 2011;95(3):915-921. doi:10.1016/j.fertnstert.2010.11.026 21130988 PMC3043157

[32] Marshall AL, Arora VM, Salles A. Physician fertility: a call to action. Acad Med. 2020;95(5):679-681. doi:10.1097/ACM.0000000000003079 31738214

[33] Juengst SB, Royston A, Huang I, Wright B. Family leave and return-to-work experiences of physician mothers. JAMA Netw Open. 2019;2(10):e1913054. doi:10.1001/jamanetworkopen.2019.13054 31603485 PMC6804274

[34] Lerner LB, Baltrushes RJ, Stolzmann KL, Garshick E. Satisfaction of women urologists with maternity leave and childbirth timing. J Urol. 2010;183(1):282-286. doi:10.1016/j.juro.2009.08.113 19913817

[35] Humphries LS, Lyon S, Garza R, Butz DR, Lemelman B, Park JE. Parental leave policies in graduate medical education: a systematic review. Am J Surg. 2017;214(4):634-639. doi:10.1016/j.amjsurg.2017.06.023 28751061

[36] Rangel EL, Castillo-Angeles M, Changala M, Haider AH, Doherty GM, Smink DS. Perspectives of pregnancy and motherhood among general surgery residents: a qualitative analysis. Am J Surg. 2018;216(4):754-759. doi:10.1016/j.amjsurg.2018.07.036 30072028

[37] MacVane CZ, Fix ML, Strout TD, Zimmerman KD, Bloch RB, Hein CL. Congratulations, you’re pregnant! now about your shifts . . . : the state of maternity leave attitudes and culture in EM. West J Emerg Med. 2017;18(5):800-810. doi:10.5811/westjem.2017.6.33843 28874931 PMC5576615

[38] Riano NS, Linos E, Accurso EC, . Paid family and childbearing leave policies at top US medical schools. JAMA. 2018;319(6):611-614. doi:10.1001/jama.2017.19519 29450516 PMC5838606

[39] Adesoye T, Mangurian C, Choo EK, Girgis C, Sabry-Elnaggar H, Linos E; Physician Moms Group Study Group. Perceived discrimination experienced by physician mothers and desired workplace changes: a cross-sectional survey. JAMA Intern Med. 2017;177(7):1033-1036. doi:10.1001/jamainternmed.2017.1394 28492824 PMC5818808

[40] Stack SW, Jagsi R, Biermann JS, . Maternity leave in residency: a multicenter study of determinants and wellness outcomes. Acad Med. 2019;94(11):1738-1745. doi:10.1097/ACM.0000000000002780 31094723

[41] Altieri MS, Salles A, Bevilacqua LA, . Perceptions of surgery residents about parental leave during training. JAMA Surg. 2019;154(10):952-958. doi:10.1001/jamasurg.2019.2985 31389989 PMC6686777

