# Supplementary material for: Knowledge of Fertility and Perspectives About Family Planning Among Female Physicians
Source: JAMA Netw Open. 2022 May 18;5(5):e2213337. doi: 10.1001/jamanetworkopen.2022.13337 (PMC9118076; doi:10.1001/jamanetworkopen.2022.13337)
Supplement: Supplement. — eAppendix 1. Qualitative Interview Guide eAppendix 2. Qualitative Interview Codebook eAppendix 3. Pilot Survey eAppendix 4. Themes and Subthemes That Arose From Qualitative Interviews With 16 Women Physicians [file jamanetwopen-e2213337-s001.pdf]

Supplemental Online Content

Smith KS, Bakkensen JB, Hutchinson AP, et al. Knowledge of fertility and perspectives about family planning among female physicians. *JAMA Netw Open.* 2022;5(5):e2213337.
doi:10.1001/jamanetworkopen.2022.13337

eAppendix 1. Qualitative Interview Guide

eAppendix 2. Qualitative Interview Codebook

eAppendix 3. Pilot Survey

eAppendix 4. Themes and Subthemes That Arose From Qualitative Interviews With 16 Women Physicians

This supplemental material has been provided by the authors to give readers additional information about their work.

© 2022 Smith KS et al. *JAMA Network Open.*

eAppendix 1. Qualitative Interview Guide

Introduction:
Welcome. Thank you for taking the time to join us today. We are researchers interested in understanding the fertility and family planning experiences of female physicians, and how these experiences may influence the career paths that women take. For this study, we are interested in interviewing female physicians who are currently in academic medicine as well as female physicians who have left academic medicine. We hope that you will share your experiences with us so that we may learn what policies and changes may better support future female physicians like you in the future. There are no right or wrong answers, because everyone experiences things differently. We are interested in the full range of perspectives and experiences, both positive and negative, so please feel free to share your point of view.

We will be audio recording today's session because we want to be able to focus on your comments and not on trying to write everything down. Afterwards, we will transcribe the recording and make sure to remove any identifying information if there is any, and after that we will delete the recording. All data will be analyzed in aggregate, no identifying information will be linked to the transcripts. Only study team members will have access to the recordings and transcripts, which will be kept on a secure server at Northwestern.

Any questions before we begin?

INTRODUCTIONS
To begin, please tell us a little bit about yourself and your career path up until this point. Where did you complete your medical school and residency? What specialty did you train in?
What is your current position?

QUESTIONS

- **Medical Education on Fertility and the Role of Age in Fertility**
  - Did you learn about the role of age in fertility in your medical training? When did you learn about it?
  - How should medical education be changed to better educate medical trainees about fertility and the role of age in fertility?
- **Workplace Support for Pregnancy and Parenthood**
  - Do you have children? If so, how many do you have?
    - <u>For those who have children:</u>
      - What stage of your career were you in when you had your child/children (e.g., medical student, resident, fellow, attending)?
      - **Parental Leave**
        - How much parental leave did you take?
        - If you were partnered at the time, how much parental leave did your partner take?
        - How satisfied were you with the amount of parental leave you took?
        - What would your ideal amount of parental leave be?
      - **Leadership Support**
        - How supportive was the leadership in your department of your pregnancy and parenthood? That is, your department head, division chief, or your program director if you were a trainee at the time.
        - In what ways were they supportive or not? Can you provide specific examples?
        - Do you think leadership support of parenthood differs depending on whether the leaders are male or female?
      - **Policies/Systems that Support Pregnancy & Parenthood**
        - How do you think academic practice differs from private practice in terms of workplace support for pregnancy and parenthood?
        - Were there ways in which your workplace could have better supported you during your experiences of pregnancy and parenthood?
        - What policies or changes could be made in the workplace to better support future female physicians in pregnancy and parenthood?
    - <u>For those who do not have children:</u>
      - **Leadership Support**

© 2022 Smith KS et al. *JAMA Network Open.*

        - How supportive do you think your departmental leadership is of pregnancy and parenthood? That is, your chair, division chief, or program director (if they are a trainee)?
        - In what ways are the leaders supportive or not? Can you provide specific examples that you have observed?
        - Do you think leadership support of parenthood differs depending on whether they are male or female?
      - **Policies/Systems that Support Pregnancy & Parenthood**
        - How do you think academic practice differs from private practice in terms of workplace support for pregnancy and parenthood?
        - What policies or changes could be made in the workplace to better support future female physicians in pregnancy and parenthood?
- **Impact of career path on family planning decisions**
  - Did you delay having children because of your medical training or career?
  - Have you ever felt concerned about how the length of your training would impact your family planning?
- **Impact of career path on your choice of partner**
  - Are you currently partnered?
    - For those who are partnered:
      - How has your partner modified his or her career path to support your training and career?
      - How has your partner modified his or her career path to accommodate family planning and parenthood?
      - When choosing a romantic partner, did you take into consideration how your partner's career could accommodate parenthood?
        - Prompt: For instance, did you take into consideration factors such as their financial stability, workload, and the flexibility in their workplace schedules when choosing a romantic partner?
    - For those who are not partnered:
      - When choosing a romantic partner, to what extent do you take into consideration how their career could accommodate parenthood?
        - Prompt: For instance, do you take into consideration factors such as their financial stability, workload, and the flexibility in their workplace schedules when choosing a romantic partner?
      - Have you ever considered having children without a romantic partner?
- **Impact of family planning on career path**
  - Have you ever considered taking an extended leave (> 12 weeks) from your medical training or career for childbearing and parenthood?
  - Have you ever considered choosing a different subspecialty or career to accommodate childbearing and parenthood?
  - Have you ever considered reducing your work hours to accommodate childbearing and parenthood?
  - Have you ever considered leaving medicine altogether to accommodate childbearing and parenthood?
  - For those who have children:
    - How has your career been impacted by parenthood?
    - If you are partnered, how has your partner's career been impacted by parenthood?
- **Factors that Influence Decision Making about Egg Freezing**
  - Have you ever considered freezing your eggs for fertility preservation?
    - What are the primary factors that you would consider when making a decision about whether or not to freeze your eggs?
    - **Personal Factors:**
      - How much would your relationship status factor into your decision about whether or not to freeze your eggs?
      - How much would your age factor into your decision about whether or not to freeze your eggs?
    - **Financial Cost/Insurance Coverage:**
      - How much would the financial cost of egg freezing factor into your decision about whether or not to freeze your eggs?
      - Are you aware of whether fertility preservation is covered by your health insurance?
        - If so, are you aware of the approximate out-of-pocket costs and copays that are associated with the fertility preservation procedures?
      - If you learned that fertility preservation was covered by your health insurance, would you use it?
      - If fertility preservation was not covered by your health plan, how much would you be willing to pay to get that coverage?
    - **Workplace Support:**
      - How much would the time associated with fertility preservation procedures factor into your decision about whether or not to freeze your eggs?
      - How supportive would your departmental leadership be if you asked for flexibility in your work schedule to undergo fertility preservation?

© 2022 Smith KS et al. *JAMA Network Open.*

            - How supportive would your departmental leadership be if you asked for flexibility in your work schedule to undergo infertility treatment?
- **What Are You Planning to Do/What Would You Do Differently?**
    - If you were to counsel a young female trainee on how to navigate family planning and career decisions, what advice would you give?
    - <u>For those who have not attempted to have children:</u>
        - What are some of the ways in which you plan to balance your family planning and career decisions moving forward?
        - **Changing Timing of Family Planning**
            - Would you consider attempting to conceive soon, even if the timing is not optimal for your training or your career?
            - Would you consider freezing your eggs to extend fertility?
            - Would you consider delaying trying to conceive until later, when the timing may be more optimal for your training or your career?
        - **Changing Career Path**
            - Would you consider taking an extended leave from medical training or your career for childbearing?
            - Would you consider going into a different specialty or career to accommodate parenthood?
    - <u>For those who have attempted to have children:</u>
        - If you could do it over again, what would you do differently regarding your family planning and career decisions?
        - **Changing Timing of Family Planning**
            - Would you attempt to conceive earlier?
            - Would you start trying to conceive later?
            - Would you freeze your eggs to extend fertility?
        - **Changing Career Path**
            - Would you start your medical training earlier?
            - Would you take an extended leave (> 12 weeks) from medical training or your career to accommodate parenthood?
            - Would you choose not to take an extended leave from medical training or your career to accommodate parenthood?
            - Would you go into a different specialty or career?

Final Thoughts
- Of all the things we discussed today, what do you consider the most important?
- Is there anything else you would like to share about your experiences with fertility and family planning in your medical career?
- Thank you. Your input is key to helping us understand what changes and policies we can make to better support female physicians in the future. We are so grateful that you agreed to take the time to join our discussion today.

eAppendix 2. Qualitative Codebook

Medical Education/Awareness on the Role of Age in Fertility
Operational definition: Women's education, awareness, and understanding on the role of age in fertility and how age influences the likelihood of success for assisted reproductive technology (ART), including egg freezing, embryo freezing, and IVF. Code any segments of the transcripts that include discussion of women's awareness, education, and understanding around the role of age in fertility and how age influences the likelihood of ART success.
Examples include:
- Education/training/awareness on the role of age in fertility and infertility
- Education/training/awareness on how age influences likelihood of ART success
- Education/training awareness on topics including miscarriages, obstetric complications etc.
- Understanding the risks of ART (risk of the procedure, financial risks, emotional risks)
- Sources of knowledge/awareness:
    - Medical education/training, personal experience, private discussions with friends, colleagues/peers, mentors, departmental leadership
- Emotional impact of infertility, miscarriages, and complications
    - Feelings of shame, devastation, stigma, isolation
- Workshops/training to discuss options surrounding fertility, infertility and family planning
- Education/awareness targeted for female physicians/trainees
    - Education/training around procreative management in residency/early career faculty
    - Rates of infertility among female physicians compared with general population

© 2022 Smith KS et al. *JAMA Network Open.*

  - Considerations of the role of age in fertility when having <u>multiple children</u>
  - Discussion on the emotional and financial aspects of infertility treatment

Financial Cost and Insurance Coverage
Operational definition: The financial cost of fertility preservation, infertility treatment, and childbearing. This code also focuses on insurance coverage for fertility preservation and/or infertility treatment, and how this coverage can influence women's decisions. Code any segments of the transcripts that include discussion of financial costs and insurance coverage, and how it plays a role in family planning, career, fertility preservation, and infertility treatment decisions.
Examples include:

- Financial debt from medical training
- Financial stability from the participant's own income
- Financial stability from romantic partner's income or financial support from family members
- Financial considerations for assisted reproductive technology (e.g., egg freezing, IVF)
- Cost of child rearing (e.g., cost of childcare/daycare, sick care, schools)
- Insurance coverage for fertility preservation and infertility treatment
- How people learn about their insurance benefits for fertility, infertility, family planning

Workplace Support for Work-Life Balance (Support Inside Work)

Operational definition: The support that participants receive inside the workplace for work-life balance, family planning and parenthood. Code any segments of the transcripts that include discussion of <u>formal mechanisms of support in the workplace</u> (e.g., work schedules, parental leave policies) as well as <u>informal mechanisms of support in the workplace</u> (e.g., leadership support, support from colleagues, norms about families in department) and how it may play a role in family planning, career, and fertility preservation decisions.

Examples include:

1) **Formal Mechanisms of Support in the Workplace**
   - Schedules
     - Control and flexibility in scheduling and/or changing schedules
     - The amount of coverage/support/redundancy they have in their role/department for taking time off (e.g., hospitalists tend to feel a greater sense of control/flexibility in determining their work schedule)
     - Extent to which changes in scheduling (e.g., taking time off for parental leave) burdens others (partners in their practice, co-residents, colleagues)
     - How different roles (e.g., research, teaching, clinical) offer more or less flexibility in scheduling
     - How flexibility and control in scheduling can impact decisions around fertility preservation and ART
   - Policies and resources for supporting parenthood in a department/division/practice
     - Lactation support (e.g., policies, lactation rooms/pods, pumps, flexibility)
     - Parental leave policies
     - On-site childcare
     - Backup childcare/sick care
     - Support groups for female physician moms
     - Mentorship/panels on work-life balance and family planning
2) **Informal Mechanisms of Support in the Workplace**
   - Norms in the department around family planning and childrearing
   - How supported women feel/felt taking time off work for parental leave
   - Leadership support: Departmental leaders' support of families and parenthood (e.g., explicitly supportive, not supportive/obstructive, more distant/out of touch)
     - Whether leadership support differs by the gender of the leaders (e.g., supportiveness of male vs. female leaders)
   - Perceptions of workplace support surrounding infertility
   - Perceptions of workplace support surrounding fertility preservation
   - Supportiveness of colleagues
   - Career advice to pick more flexible/family-friendly career paths
   - Career advice on how others have done it well

Romantic Partner, Family, Network Support (Support Outside Work)

Operational definition: The financial, emotional, and instrumental support that women receive outside of work (from their romantic partners, family members, and social network) to support work-life balance. Code any segments of the transcripts that include discussion of support (or lack of support) received from romantic partners, family members, network members and how it may play a role family planning, career, fertility preservation, and infertility treatment decisions.

© 2022 Smith KS et al. *JAMA Network Open.*

Examples include:

- Presence/absence of romantic partner
- Extent to which the participant accommodated her career for her partner's career
- Extent to which the partner accommodated his/her career to support participant's career
- Extent to which the participant accommodated her career for family planning/parenthood
- Extent to which the romantic partner accommodated his or her career for family planning/parenthood
- Presence/absence of family members of other network members to support family planning and parenthood (e.g., family members or friends nearby that can help out with child care, financial support from family members to help out with family planning and/or fertility preservation)

Influence of Fertility and Family Planning on Career Path

Operational definition: The ways in which fertility and family planning decisions influenced the career paths that women take, including their specialty selection and/or choices that influence long-term career trajectory. Code any segments of the transcripts that include discussion of how fertility and family planning influenced women's decisions surrounding women's career paths, including specialty selection, going into academic medicine vs. private practice, choosing hospital medicine, going part time, and any concerns women may have about how fertility/family planning may influence their career performance/trajectory.

Examples include:

- Whether fertility/family planning influenced specialty selection
- Whether fertility/family planning influenced the type of practice they went into/plan to go into (e.g., academic medicine, private practice, community)
- Whether fertility/family planning influenced decisions to go part time
- Whether fertility/family planning influenced
- Concerns about how fertility/family planning may influence career performance/trajectory

Influence of Fertility and Family Planning on Career Path

Operational definition: The ways in which fertility and family planning decisions influenced the career paths that women take, including their specialty selection and/or choices that influence long-term career trajectory. Code any segments of the transcripts that include discussion of how fertility and family planning influenced women's decisions surrounding women's career paths, including specialty selection, going into academic medicine vs. private practice, choosing hospital medicine, going part time, and any concerns women may have about how fertility/family planning may influence their career performance/trajectory.

Examples include:

- Delaying child-bearing because of length of medical training/career
- Choosing a different specialty/career path (e.g., hospital medicine) that is family friendly
- Choosing to go part time or reduce work hours to accommodate parenthood
- Choosing to go into academic vs private practice for family planning/parenthood concerns

eAppendix 3. Pilot Survey Instrument

What is your date of birth (mm/dd/yyyy)?

____

What is your current gender identity?

© 2022 Smith KS et al. *JAMA Network Open.*

- Male
- Female
- Non-binary
- Genderfluid
- Not listed (please specify) ____
- Prefer not to answer

What was your assigned sex at birth?

- Male
- Female

What is your sexual orientation?

- Heterosexual (or straight)
- Gay or lesbian
- Bisexual
- Not listed (please specify) ____
- Prefer not to answer

With which race/ethnicity do you identify? (Please check all that apply)

- White
- Black or African American
- Asian
- American Indian or Alaska Native
- Hispanic or Latino
- Native Hawaiian or Other Pacific Islander
- Middle Eastern or North African
- Multiracial
- Not Listed (please specify) _______________
- Prefer not to answer

Which of these categories best describes your total combined household income for the past 12 months?

- < $99K
- $100K - $250K
- $250K - $500K
- > $500K
- Don't know
- Prefer not to answer

What is your current relationship status?

- Married/Partnered
- Single
- Divorced/Separated
- Widowed
- Not Listed (please specify) ____

How many hours a week do you work?

- 1 to 19 hours
- 20 to 39 hours
- 40 to 59 hours
- > 60 hours/week

© 2022 Smith KS et al. *JAMA Network Open.*

- ☐ Not employed
- ☐ Furloughed
- ☐ Retired
- ☐ Not Listed (please specify) ____

Display This Question:
If What is your current relationship status? = Married/Partnered

How many hours a week does your spouse/partner work?

- ☐ 1 to 19 hours
- ☐ 20 to 39 hours
- ☐ 40 to 59 hours
- ☐ > 60 hours/week
- ☐ Not employed
- ☐ Furloughed
- ☐ Retired
- ☐ Not Listed (please specify) ____

On a scale from 0-100%, please allocate how the responsibilities for maintaining your household and family life are distributed in your household. If the situation is not applicable to you, please allocate 100% to the "Not Applicable" option.

	You	Spouse/Partner	Paid Family Member/ Other	Unpaid Family Member/ Other	Not Applicable
Household maintenance (e.g., cleaning, groceries, cooking, laundry)	___%	___%	___%	___%	___%
Family maintenance (e.g., childcare, elder care, healthcare appointments, school forms)	___%	___%	___%	___%	___%

In what year did you graduate medical school?

____

Are you currently practicing medicine?

- ☐ Yes
- ☐ No

What is your current position?

- ☐ Resident
- ☐ Fellow
- ☐ Attending Physician
- ☐ Not Listed (please specify) ____

In what specialty/specialties have you trained?

____

Display This Question:
If What is your current position? = Attending physician

What type of practice setting do you currently work in? Please check all that apply.

- ○ Academic practice
- ○ Private practice
- ○ Community practice
- ○ Not Listed (please specify) ____

© 2022 Smith KS et al. *JAMA Network Open.*

Display This Question:
If What is your current position? = Resident
Or What is your current position? = Fellow

What type of practice setting do you plan to go into? Please check all that apply.

- ○ Academic practice
- ○ Private practice
- ○ Community practice
- ○ Not Listed (please specify) ____

I feel burned out from my work.

- ▢ Never
- ▢ A few times a year
- ▢ Once a month or less
- ▢ A few times a month
- ▢ Once a week
- ▢ A few times a week
- ▢ Every day

I have become more callous toward people since I took this job.

- ▢ Never
- ▢ A few times a year
- ▢ Once a month or less
- ▢ A few times a month
- ▢ Once a week
- ▢ A few times a week
- ▢ Every day

Do you have children (e.g., biological, adopted, step, or foster children)?

- ☐ Yes
- ☐ No
- ☐ Prefer not to answer

Display This Question:
If Do you have children (e.g., biological, adopted, step, or foster children)? = Yes

How many biological children do you have?

- ○ 0
- ○ 1
- ○ 2
- ○ 3
- ○ 4
- ○ 5+

© 2022 Smith KS et al. *JAMA Network Open.*

Display This Question:
If Do you have children (e.g., biological, adopted, step, or foster children)? = Yes

How many adopted, step, or foster children do you have?

- 0
- 1
- 2
- 3
- 4
- 5+

Display This Question:
If Do you have children (e.g., biological, adopted, step, or foster children)? = Yes

Do you intend to have additional children in the future? (e.g., biological, adopted, step, or foster)?

- Yes
- No
- Undecided
- Prefer not to answer

Display This Question:
If Do you intend to have additional children in the future? (e.g., biological, adopted, step, or fos... = Yes

How many additional biological children do you plan to have in the future?

- 0
- 1
- 2
- 3
- 4
- 5+

Display This Question:
If Do you have children (e.g., biological, adopted, step, or foster children)? = No

Do you intend to have children in the future? (e.g., biological, adopted, step, or foster)?

- Yes
- No
- Undecided
- Prefer not to answer

© 2022 Smith KS et al. *JAMA Network Open.*

Display This Question:
If Do you intend to have children in the future? (e.g., biological, adopted, step, or fos… = Yes

How many biological children do you plan to have in the future?

- ○ 0
- ○ 1
- ○ 2
- ○ 3
- ○ 4
- ○ 5+

Display This Question:
If What was your assigned sex at birth = Female

How many times have you been pregnant (if any)?

- ○ 0
- ○ 1
- ○ 2
- ○ 3
- ○ 4
- ○ 5+

Display This Question:
How many times have you been pregnant (if any) > = 1

What were the outcome(s) of the pregnancy/pregnancies? (Please select all that apply).

- ○ Live born
- ○ Miscarriage
- ○ Termination
- ○ Not Listed (please specify) ________________

Display This Question:
How many times have you been pregnant (if any) > = 1

Do you think your pregnancy outcome(s) or fertility were impacted by your medical training or career?

- ○ Yes
- ○ No
- ○ Undecided
- ○ Prefer not to answer

Display This Question:
Do you think your pregnancy outcome(s) was impacted by your medical training or career? = Yes

Please specify how your pregnancy outcome(s) may have been impacted by your medical training or career:

____

© 2022 Smith KS et al. *JAMA Network Open.*

Did you delay having children because of your medical training or career? (Please select all that apply).

- ○ Yes, I have delayed in the past
- ○ Yes, I am currently still delaying
- ○ No
- ○ Other (please specify) ______________
- ○ Prefer not to answer

Display This Question:
If Did you delay having children because of your medical training or career? = Yes, I delayed in the past OR
If Did you delay having children because of your medical training or career? = Yes, I am currently still delaying

How long did you delay (or plan to delay) having children?

- ○ 0 to 3 years
- ○ 3 to 5 years
- ○ 5+ years

Did the following factors influence your decisions about the timing of childbearing?

	Not at all	A little bit	Moderately	Very Much	Extremely
Lack of time	○	○	○	○	○
Lack of flexibility in schedule	○	○	○	○	○
Financial strain	○	○	○	○	○
Lack of romantic partner	○	○	○	○	○
Lack of support from colleagues	○	○	○	○	○
Concern about burdening colleagues with extra work	○	○	○	○	○
Reputational concerns (e.g., being perceived as less committed to career	○	○	○	○	○
Lack of support from leadership (e.g., chair, division chief, practice manager)	○	○	○	○	○
Lack of social support nearby (e.g., extended family, friends)	○	○	○	○	○
Stress	○	○	○	○	○
Not ready for children	○	○	○	○	○

Have you ever felt concerned about how the length of your medical training would impact your family planning?

- ☐ Not at all concerned
- ☐ A little bit concerned
- ☐ Moderately concerned
- ☐ Very concerned
- ☐ Extremely concerned

Have you done the following to accommodate childbearing or parenthood?

	Yes	No	Not Applicable
Taken an extended leave (over 12 weeks) from your medical training or career to accommodate childbearing or parenthood	○	○	○
Chosen a different specialty or career to accommodate childbearing or parenthood	○	○	○
Reduced your work hours to accommodate childbearing or parenthood	○	○	○
Left medicine to accommodate childbearing or parenthood	○	○	○
Changed the setting of your work (e.g., academic vs. private practice)	○	○	○
Not taken opportunities for career advancement	○	○	○

Display This Question Block:
If What was your assigned sex at birth = Female

Have you ever considered egg/embryo freezing for fertility preservation?

○ Yes
○ No
○ Prefer not to answer

Display This Question:
If Have you ever considered egg/embryo freezing for fertility preservation? = Yes

Did you seek consultation for egg/embryo freezing at a fertility center?

○ Yes
○ No
○ Other (please specify) ____
○ Prefer not to answer

Display This Question:
If Have you ever considered egg/embryo freezing for fertility preservation? = Yes

Did you freeze your eggs/embryos?

○ Yes
○ No
○ Other (please specify) ____
○ Prefer not to answer

Display This Question:
Did you freeze your eggs/embryos = Yes

At what age did you freeze your eggs/embryos?

____

Display This Question:

Did you freeze your eggs/embryos = No

Or Did you freeze your eggs/embyros = Other

Do you plan to freeze your eggs in the future?

- ○ Yes
- ○ No
- ○ Don't know
- ○ Other (please specify) ____
- ○ Prefer not to answer

Display This Question:

Do you plan to freeze your eggs in the future = Yes

At what age do you plan to freeze your eggs/embryos?

____

Is egg/embryo freezing covered by your health insurance?

- ○ Yes
- ○ No
- ○ Don't know

Are you aware of the approximate out-of-pocket cost that is associated with freezing your eggs/embryos?

- ○ Yes
- ○ No

If you learned that egg/embryo freezing was covered by your health plan, would you (or your partner) use it?

- ○ Yes
- ○ No
- ○ Not sure

If egg/embryo freezing was not covered by your health plan, how much would you be willing to pay out of pocket to freeze your (or your partner's) eggs/embryos?

- ○ $0
- ○ $1 to $999
- ○ $1000 to $4,999
- ○ $5000 to $9,999
- ○ $10,000 to $19,999
- ○ $20,000+

© 2022 Smith KS et al. *JAMA Network Open.*

Display This Question:
If Have you ever considered freezing your eggs/embryos for fertility preservation? = Yes

Did the following factors influence your initial consideration of freezing your eggs/embryos or your decision about whether or not to go through the process of freezing your eggs/embryos?

	Not at all	A little bit	Moderately	Very Much	Extremely	Not Applicable
Relationship status	○	○	○	○	○	○
Age	○	○	○	○	○	○
Financial cost	○	○	○	○	○	○
Insurance coverage	○	○	○	○	○	○
Time commitment	○	○	○	○	○	○
Potential risks associated with procedure	○	○	○	○	○	○
Efficacy/likelihood of success	○	○	○	○	○	○
Burden (physical and emotional) associated with procedure	○	○	○	○	○	○

Display This Question Block:
If What was your assigned sex at birth = Female

Have you ever experienced infertility?

- ○ Yes
- ○ No
- ○ Have not tried to conceive
- ○ Not applicable
- ○ Prefer not to answer

Display This Question:
If Have you ever experienced infertility? = Yes

Have you ever used in vitro fertilization (IVF) for conception?

- ○ Yes
- ○ No
- ○ Not applicable
- ○ Prefer not to answer

Display This Question:
If Have you ever used in vitro fertilization (IVF) for conception? = Yes

At what age did you undergo in vitro fertilization (IVF)?

© 2022 Smith KS et al. *JAMA Network Open.*

Thank you for sharing your personal experiences. We are also interested in assessing female physicians' knowledge of fertility and fertility preservation and the sources that female physicians rely on to learn this knowledge. We hope this information may help inform our understanding of how to better educate medical trainees on the role of age in fertility and fertility preservation.

Over which age range does a woman's ability to get pregnant decline most precipitously?

○ 25 to 29 years old

○ 30 to 34 years old

○ Over 35 years old

For each age range, please indicate how ideal it would be for a woman to freeze her eggs.

	Not at all ideal 0	1	2	3	Extremely ideal 4
25 to 29 years old	○	○	○	○	○
30 to 34 years old	○	○	○	○	○
35 to 37 years old	○	○	○	○	○
37 to 39 years old	○	○	○	○	○
40 to 44 years old	○	○	○	○	○

What is the likelihood of pregnancy in a month with properly timed intercourse with normal semen analysis if a female is:

	<5%	10%	15%	25%	40%	60%	80%
30 to 34 years old	○	○	○	○	○	○	○
35 to 39 years old	○	○	○	○	○	○	○
40 to 43 years old	○	○	○	○	○	○	○
43 to 45 years old	○	○	○	○	○	○	○

What is the likelihood of live birth for a single In-vitro Fertilization (IVF) cycle for a woman at each of the following ages using her own eggs:

	<5%	10%	15%	25%	40%	60%	80%
30 to 34 years old	○	○	○	○	○	○	○
35 to 39 years old	○	○	○	○	○	○	○
40 to 43 years old	○	○	○	○	○	○	○
43 to 45 years old	○	○	○	○	○	○	○

To what extent have you relied on the following sources to learn about the role of age in fertility?

	Not at all	A little bit	Moderately	Very Much	Extremely
Formal education in medical school	○	○	○	○	○
Your own healthcare provider (e.g., OBGYN, PCP)	○	○	○	○	○
Your own personal experiences	○	○	○	○	○
Experiences of family and friends	○	○	○	○	○
Experiences of patients	○	○	○	○	○
Experiences of female physician colleagues	○	○	○	○	○
Internet	○	○	○	○	○
News outlets	○	○	○	○	○
Television/film	○	○	○	○	○

© 2022 Smith KS et al. *JAMA Network Open.*

Individual posts from social media (e.g., Instagram, Twitter, Facebook)	○	○	○	○	○
Educational content from social media (e.g., Instagram, Twitter, Facebook)	○	○	○	○	○

To what extent have you relied on the following sources to learn about elective egg freezing?

	Not at all	A little bit	Moderately	Very Much	Extremely
Formal education in medical school	○	○	○	○	○
Your own healthcare provider (e.g., OBGYN, PCP)	○	○	○	○	○
Your own personal experiences	○	○	○	○	○
Experiences of family and friends	○	○	○	○	○
Experiences of patients	○	○	○	○	○
Experiences of female physician colleagues	○	○	○	○	○
Internet	○	○	○	○	○
News outlets	○	○	○	○	○
Television/film	○	○	○	○	○
Individual posts from social media (e.g., Instagram, Twitter, Facebook)	○	○	○	○	○
Educational content from social media (e.g., Instagram, Twitter, Facebook)	○	○	○	○	○

If you could do it over again, would you do have done the following differently regarding your family planning and career decisions?

	Strongly Disagree	Disagree	Neutral	Agree	Strongly Agree	Not Applicable
Attempt to conceive earlier	○	○	○	○	○	○
Start trying to conceive later	○	○	○	○	○	○
Freeze your eggs to extend fertility	○	○	○	○	○	○
Take an extended leave (over 12 weeks) from your medical training or career to accommodate childbearing or parenthood	○	○	○	○	○	○
Choose a different specialty or career to accommodate childbearing or parenthood	○	○	○	○	○	○
Reduce your work hours to accommodate childbearing or parenthood	○	○	○	○	○	○
Leave medicine to accommodate childbearing or parenthood	○	○	○	○	○	○

© 2022 Smith KS et al. *JAMA Network Open.*

eAppendix 4. Themes and subthemes that arose from qualitative interviews with 16 women physicians

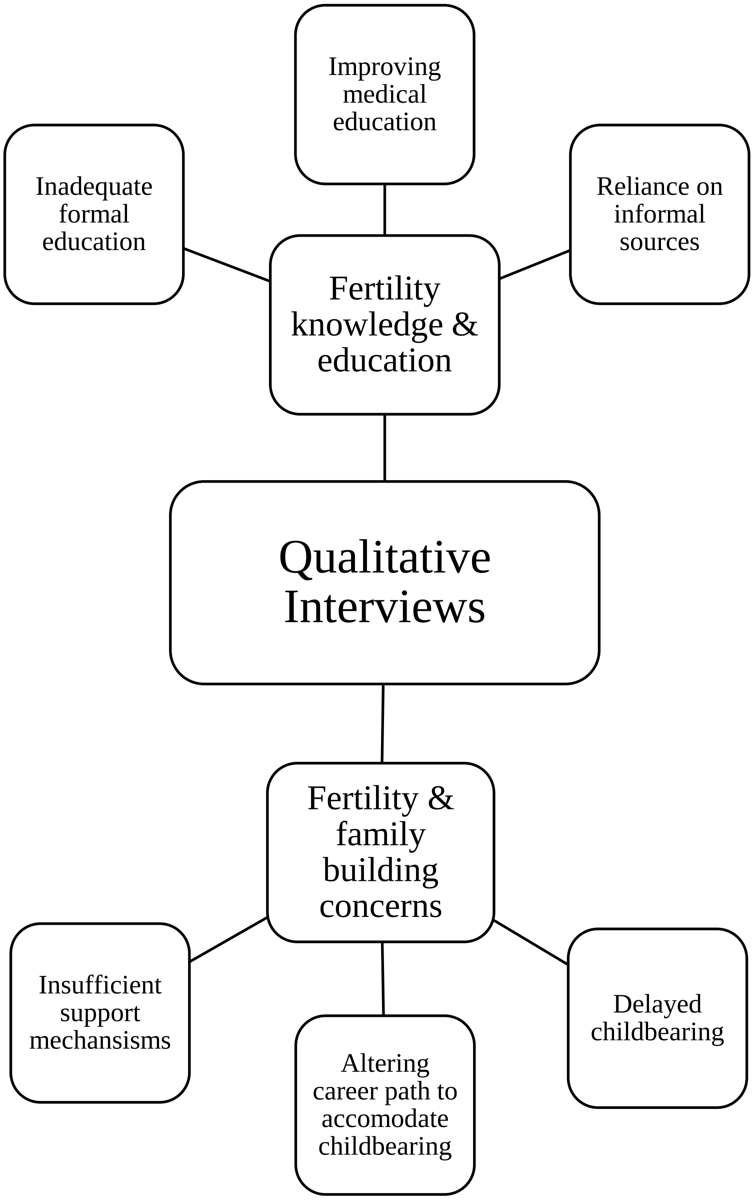

© 2022 Smith KS et al. *JAMA Network Open.*
